# Understanding Metabolic Remodeling in *Mycobacterium smegmatis* to Overcome Energy Exigency and Reductive Stress Under Energy-Compromised State

**DOI:** 10.3389/fmicb.2021.722229

**Published:** 2021-09-01

**Authors:** Varsha Patil, Vikas Jain

**Affiliations:** Microbiology and Molecular Biology Laboratory, Department of Biological Sciences, Indian Institute of Science Education and Research, Bhopal, India

**Keywords:** mycobacteria, lipid metabolism, triacylglycerol, ATP synthase, lipid bodies, redox regulation

## Abstract

Mycobacteria such as *Mycobacterium tuberculosis*, the causative agent of tuberculosis that annually kills several million people worldwide, and *Mycobacterium smegmatis*, the non-pathogenic fast-growing mycobacteria, require oxidative phosphorylation to meet their energy requirements. We have previously shown that deletion of one of the two copies of *atpD* gene that codes for the ATP synthase β-subunit establishes an energy-compromised state in *M. smegmatis*. Here we report that upon such deletion, a major routing of electron flux occurs through the less energy-efficient complexes of its respiratory chain. Δ*atpD* bacterium also shows an increased reduced state which is further confirmed by the overexpression of WhiB3, a major redox sensor. We show a substantial modulation of the biosynthesis of cell wall associated lipids and triacylglycerol (TAG). An accumulation of TAG-containing lipid bodies is further confirmed by using ^14^C oleate incorporation. Interestingly, the mutant also shows an overexpression of TAG-degrading lipase genes, and the intracellular lipolytic enzymes mediate TAG hydrolysis for their utilization as energy source. We believe that our *in vitro* energy-depleted model will allow us to explore the critical link between energy metabolism, redox homeostasis, and lipid biosynthesis during ATP-depleted state, which will enhance our understanding of the bacterial adaptation, and will allow us to identify novel drug targets to counter mycobacterial infections.

## Introduction

Tuberculosis, caused by the bacterium *Mycobacterium tuberculosis*, is one of the serious public health concerns globally (Eisinger et al., 2020). *M. tuberculosis* is known to reside in a dormant state for prolonged period, and targeting such dormant *M. tuberculosis* is a big challenge for the current chemotherapeutic approaches (Gengenbacher and Kaufmann, 2012). Thus, a continuous arms race between human and mycobacteria is leading to the discovery and development of novel drug targets and therapeutics against *M. tuberculosis*.

Oxidative phosphorylation (OXPHOS) is an efficient pathway to sustain bacterial growth and survival (Bald et al., 2017). During this process, electrons obtained through the donors of central metabolic pathways are transferred from the respiratory complexes to O_2_ *via* electron transport chain (ETC) (Matsoso et al., 2005). This highly flexible respiratory chain in mycobacteria consist of various membrane-embedded electron carrier molecules and enzymes that mediate the transfer of electron, which is coupled with the generation of proton motive force (PMF) that drives ATP synthesis (Cook et al., 2014). The electron travels from complex-I (NADH dehydrogenase) to complex-II (succinate dehydrogenase) with the help of electron carrier molecule menaquinone. The chain is terminated *via* two terminal oxidases namely *aa3*-type cytochrome *c* oxidase (supercomplex along with cytochrome *bc1* and cytochrome *c*) and cytochrome *bd* oxidase which eventually catalyzes the reduction of O_2_ to water molecule by using the electrons from menaquinone and cytochrome *c*, respectively (Kana et al., 2001; Matsoso et al., 2005). Previous studies suggest that in *Mycobacterium smegmatis* during low growth rate and low air saturation (0.6%), the cytochrome *bd* oxidase complex is upregulated >50-fold, which exemplifies the importance of cytochrome *bd* at low O_2_ rates (Berney and Cook, 2010). Moreover during hypoxia under the inhibitory conditions of cytochrome *bc1-aa3* oxidase complex, cytochrome *bd* has been shown to play an important role in cell survival (Kana et al., 2001; Cook et al., 2014). Furthermore, the involvement of cytochrome *bd* in adaptation of *M. tuberculosis* toward adverse environmental conditions is very well known (Giuffre et al., 2012; Forte et al., 2016). Hence, respiration plays a very important role in mycobacterial survival in order to respond and adapt toward different environmental niche and stress conditions.

The inhibition of respiratory chain may occur either by suppression of the respiratory complexes (Lamprecht et al., 2016; Moosa et al., 2017) or due to an exposure to external stress factors such as hypoxia, nutrient deficiency, acid, NO, CO, etc., which eventually influence the redox environment of the cell by making the electron carriers more reduced (Cook et al., 2014; Iqbal et al., 2018; Akela and Kumar, 2021). Hypoxia is known to induce reactive oxygen species (ROS) (Brunelle et al., 2005), whereas NO acts as one of the major antimicrobial stress agents generating reactive nitrogen species (RNS) in the cell, thereby leading to the fluctuations in redox state (Mehta and Singh, 2019). This necessitates mycobacteria to respond toward the environmental variations to maintain homeostasis. In order to defend from the internal oxidants and reductants, mycobacteria has evolved with specific mechanisms that assist in maintaining an appropriate redox balance within the cell (Zhai et al., 2019). In *M. tuberculosis*, for example, it has been suggested that during reduced O_2_ conditions, WhiB3 senses the changes in the intracellular redox environment leading to metabolic switchover to fatty acids as the preferred nutrient source for the cell (Mehta and Singh, 2019). The presence of 4Fe–4S cluster in WhiB3 helps in maintaining redox homeostasis *via* regulating the metabolism and polyketide biosynthesis in the cell, thereby assisting in the persistence of the pathogen within the host (Mehta and Singh, 2019). The metabolic dependency of *M. tuberculosis* upon host fatty acids and cholesterol ensures a long-term survival during the persistence phase of infection (Gomez and McKinney, 2004; Pandey and Sassetti, 2008). Nevertheless, how these fatty acids are acquired, stored, and further utilized is still unclear in mycobacteria.

We have previously shown that the deletion of one of the two copies of *atpD* gene that codes for the β-subunit of the ATP synthase machinery renders *M. smegmatis* in an energy-compromised state (Patil and Jain, 2019). In the present work, we investigated in detail the mechanism involved in this adaptation and report that in *M. smegmatis*, deletion of *atpD* results in the remodeling of the ETC with utilizing less efficient proton non-translocating complexes. This results in the establishment of a reductive stress in the cell, which is dissipated by a major redox sensor, WhiB3-mediated modulation of the biosynthesis of storage lipids (triacylglycerol). We have previously shown that in Δ*atpD*, β-oxidation is enhanced for the generation of energy (Patil and Jain, 2019). It, therefore, appears that *M. smegmatis* follows two distinctive and seemingly opposite pathways in order to adapt to a low-energy state. Our work here thus reveals a distinct relationship between lipid accumulation and its utilization in *M. smegmatis* under energy-compromised state, and that a respiratory shift drives the global cellular changes leading to redox imbalance and metabolic dependency on fatty acid. We believe that understanding these cellular characteristics and their modulation to stress is vital for the identification and development of novel therapeutic drug targets to treat mycobacterial infections.

## Results

We have previously shown that *M. smegmatis atpD* gene (MSMEG_4936), which codes for the β-subunit of F_0_F_1_ ATP synthase, is important for mycobacterial physiology and metabolism (Patil and Jain, 2019), and that the deletion of one of the two copies of *atpD* renders the bacterium in an energy-compromised state. In this manuscript, we explore the impact of this deletion on the physiology of *M. smegmatis* and address how cell adapts to this physiological state and survives.

### Deletion of *atpD* Results in Altered Expression of the Respiratory Chain Complexes

**FIGURE 1:**
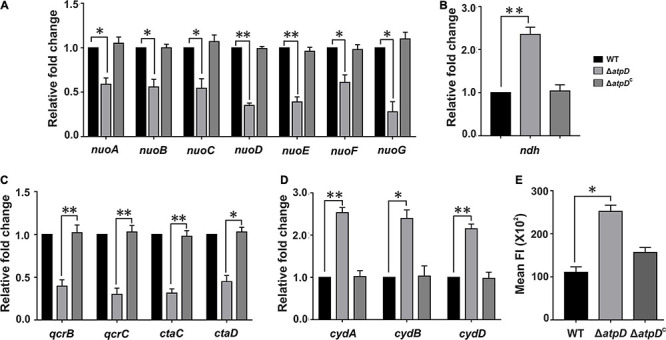
Transcriptional changes in the respiratory chain complexes and an altered membrane polarization in *M. smegmatis* Δ*atpD*. Plots show the expression profiles for genes belonging to NDH1 complex (*nuo* operon, **A**), NDH2 complex (*ndh*, **B**), cytochrome *bc1-aa3* complex (*qcr* and *cta*, **C**), and cytochrome-*bd* oxidase complex (*cyd*, **D**) in *M. smegmatis* wild-type (WT), *atpD* knock out (Δ*atpD*), and *atpD*-complemented (Δ*atpD*^*C*^) strains. Panel **(E)** shows the membrane polarization in all the three strains as monitored by DiBAC_4_(3) dye. In panels **(A–D)**, the transcript level in each case is compared with that of *M. smegmatis* WT, which is taken as 1. In all the panels, data represent an average of at least three independent experiments with error bars representing standard deviation. ^∗^*P*-value < 0.05; ^∗∗^*P*-value < 0.01.

To understand in detail how *M. smegmatis* adapts to and survive in the depleted energy state, we first looked into the highly flexible mycobacterial ETC and its complexes that facilitate mycobacterial survival by coordinating with the central metabolism under variety of environmental conditions. *M. smegmatis* consists of energy efficient type-I NADH dehydrogenase complex (NDH1), which is proton pumping in nature, encoded by *nuo* operon, and a proton non-pumping type-II NADH dehydrogenase complex (NDH2) encoded by *ndh* gene (MSMEG_3621). The two terminal oxidases utilized by mycobacterial ETC for reduction of oxygen include the energy-efficient cytochrome *bc_1_-aa_3_* supercomplex and the energetically less efficient cytochrome *bd* oxidase (Cook et al., 2014; Iqbal et al., 2018). The transfer of electron from these terminal oxidases generates a PMF, which is then subsequently utilized for ATP synthesis *via* F_0_F_1_ ATP synthase. Transcriptional analysis shows that while the genes belonging to type-I NADH dehydrogenase are downregulated (Figure 1A), the proton non-pumping type-II NADH dehydrogenase is upregulated (Figure 1B) in the Δ*atpD* strain as compared to the wild-type and the complemented strain. Similarly, the energy efficient terminal electron acceptor cytochrome *bc_1_-aa_3_* complex is found to be transcriptionally repressed (Figure 1C), whereas the energetically less efficient cytochrome *bd* oxidase genes are found to be induced (Figure 1D). This shift in the mycobacterial respiration machinery observed here suggests a modification in the respiration from proton translocating to non-proton-translocating state in Δ*atpD*. Similar observations were also seen when *M. tuberculosis* was grown under hypoxia conditions (Shi et al., 2005), suggesting a conserved respiratory adaptation in both pathogenic *M. tuberculosis* and non-pathogenic *M. smegmatis*.

We next hypothesized that such a broad change in the ETC machinery will affect the intracellular concentration of H^+^, which will result in an acidic pH in the cytoplasm and alter cellular membrane polarization. Hence we measured the changes in the membrane polarization by using a fluorescent dye DiBAC_4_(3) [Bis (1,3-dibutylbarbituric acid) trimethine oxonol], which readily diffuses across a depolarized membrane. Interestingly, the Δ*atpD* bacterium displayed increased fluorescence suggesting alteration in the membrane polarization as compared to the wild-type and the complemented strain (Figure 1E). Overall our data suggest that due to the deletion of single copy of *atpD*, the respiratory functions and energy production factors are maintained at a low level and result in altered membrane polarization.

### Altered Membrane Polarization Disrupts Redox Homeostasis in Δ*atpD*, and Is Balanced by Lipid Biosynthesis

**FIGURE 2:**
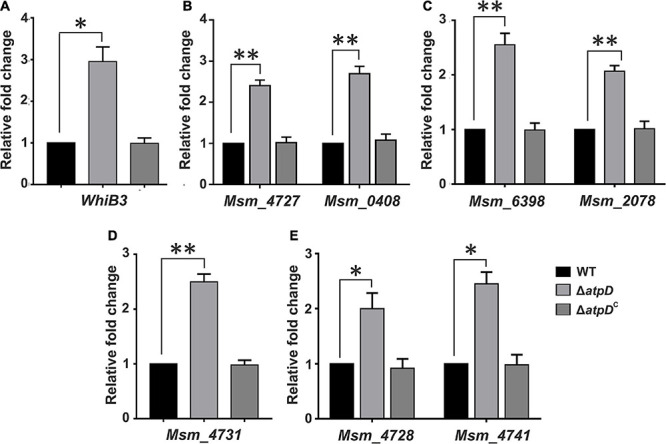
*Mycobacterium smegmatis* Δ*atpD* shows redox homeostasis under energy-deprived state. Shown are the relative mRNA transcript levels of redox sensor gene WhiB3 **(A)**, polyketide biosynthesis genes **(B)**, trehalose di-mycolate (TDM) biosynthesis genes **(C)**, polymethyl-branched fatty acid gene **(D)**, and polyketide synthase-associated protein (Pap) and *mmpL* genes **(E)** in *M. smegmatis* wild-type (WT), *atpD* knock out (Δ*atpD*), and *atpD*-complemented (Δ*atpD*^*C*^) strains. In all the panels, the transcript level in each case is compared with that of *M. smegmatis* WT, which is taken as 1. Data represent an average of at least three independent experiments with error bars representing standard deviation. ^∗^*P*-value < 0.05; ^∗∗^*P*-value < 0.01.

Membrane potential plays an important role in energy storage during OXPHOS (Zorova et al., 2018). Previous studies suggest that bacteria possess a remarkable ability to maintain their membrane polarized, even during external stress (Prindle et al., 2015; Blee et al., 2020). A sharp decline in the membrane potential is considered dangerous to the cell, due to its inefficiency to produce ATP and leading to the generation of reductive stress in the cell (Zorova et al., 2018), which leads to slow down of respiratory complexes (Akela and Kumar, 2021). Furthermore, we have previously shown that MsmΔ*atpD* has reduced levels of ATP with increased ROS and high NADH/NAD^+^ ratio, indicating an imbalanced redox state within the cell (Patil and Jain, 2019). We, therefore, asked how cell manages this intracellular redox imbalance in order to replenish the depleted NAD^+^ levels for survival. It has been suggested that *dosS/dosR* system functions as a prominent sensor for the extracellular redox signals such as reduced oxygen and NO levels (Kumar et al., 2007). WhiB3, on the other hand, is known as a major intracellular redox sensor in *M. tuberculosis*, which acts by maintaining the redox balance in the cell thereby regulating the central metabolism (Singh et al., 2009). *M. smegmatis* WhiB3 (MSMEG_1597) shares 79.8% identity with *M. tuberculosis* WhiB3 (Rv3416) (Supplementary Figure 1). Hence we hypothesized that in *M. smegmatis* under an energy-compromised state, WhiB3 acts as the major redox regulator involved in the dissipation of the reductive stress *via* synthesis of storage lipid. Therefore, we determined the expression level of WhiB3 in *M. smegmatis*, and found a threefold upregulation in its expression in *atpD* knock-out strain as compared to the wild-type and the *atpD*-complemented (*atpD*^*C*^) strain (Figure 2A). An upregulation in WhiB3 here clearly suggests that a redox state persists in *M. smegmatis* under energy-compromised state, which further corroborates with our previous study (Patil and Jain, 2019).

Furthermore, since WhiB3 in *M. tuberculosis* is known to be a positive transcriptional regulator for lipid biosynthesis genes such as those involved in polyketide biosynthesis (*pks*) and complex cell wall-associated lipids (Singh et al., 2009), we determined the mRNA expression level of MSMEG_4727 (*pks5*), which is a homolog of *M. tuberculosis pks2* with 65% identity (Supplementary Figure 2), MSMEG_0408 (*pks1*) (Figure 2B), trehalose di-mycolate (TDM) (mycolyltransferases) genes in *M. smegmatis* (Ojha et al., 2010) such as MSMEG_6398 and MSMEG_2078 (Figure 2C), MSMEG_4731, involved in the synthesis of polymethyl-branched fatty acids (Tran et al., 2019) in *M. smegmatis* (Figure 2D), and the polyketide synthase-associated protein (Pap) gene such as MSMEG_4728 (Onwueme et al., 2004; Tran et al., 2019), and *mmpL*, MSMEG_4741 (Figure 2E), which is posited to encode the enzymes involved in the synthesis and transport of glycolipids such as GPL, lipo-oligosaccharide (LOS), and sulfolipid-1 (Tran et al., 2019). Interestingly, we found an upregulation of all the cell wall-associated genes. During hypoxia, since the respiratory chain is down-regulated, *M. tuberculosis* is known to regenerate NAD^+^ from NADH *via* WhiB3, thereby sensing the intracellular redox alterations and causing the metabolic switchover to fatty acids as the preferred carbon source (Singh et al., 2009). Such metabolic shift induced by WhiB3 is known to regulate the production of methyl branched polyketides (PAT/DAT, SL-1, and PDIM) and TAG (Singh et al., 2009). Hence the observed overexpression of WhiB3 and its role in modulating polyketide biosynthesis in *M. smegmatis* Δ*atpD* immediately suggest an important function played by WhiB3 in homeostasis maintenance *via* lipid biosynthesis and as a physiological redox regulator in *M. smegmatis* under energy-compromised state.

### Loss of *atpD* Severely Affects the Cardiolipin Content in *M. smegmatis* Cell Membrane and Altered Mycolic Acid Levels Leading to Loss of Acid Fastness

**FIGURE 3:**
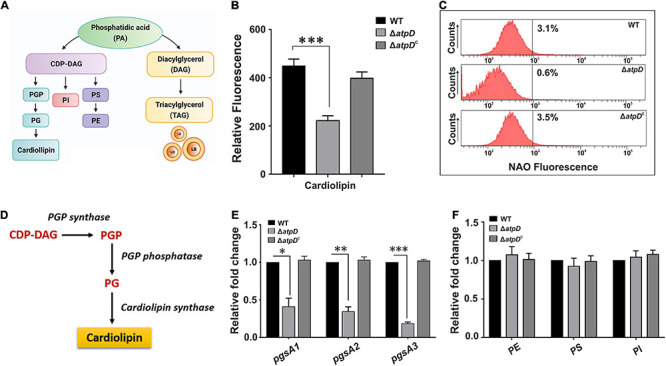
*Mycobacterium smegmatis* Δ*atpD* shows reduced cardiolipin content. Panel **(A)** shows various mycobacterial membrane lipids along with the flow of key phospholipid biosynthesis pathways. PGP, phosphatidylglycerol phosphate; PG, phosphatidylglycerol; PI, phosphatidylinositol; PS, phosphatidylserine; PE, phosphatidylethanolamine; CDP-DAG, cytidine-diphosphate diacylglycerol. Panel **(B)** shows the Nonyl Acridine orange (NAO) dye fluorescence measured for *M. smegmatis* wild-type (WT), *atpD* knock-out (Δ*atpD*), and *atpD*-complemented (Δ*atpD*^*C*^) strains. Panel **(C)** shows the flow cytometric analysis of all the three strains after staining the cells with NAO dye. The percentage value represents the fraction of cells that are positive for that fluorescence. Flow cytometry experiments were repeated at least thrice; the graph shows only one representative data. Panel **(D)** shows the cardiolipin biosynthesis pathway in mycobacteria. Panel **(E)** shows the gene expression analysis for plasma membrane biosynthesis genes *pgsA1, pgsA2*, and *pgsA3* by RT-qPCR in *M. smegmatis* wild-type, Δ*atpD*, and Δ*atpD*^*C*^ strains. Panel **(F)** shows the relative expression level of major structural genes – *MSMEG_6851* (phosphatidylethanolamine or PE), *MSMEG_0860* (phosphatidylserine or PS), and *MSMEG_2933* (phosphatidylinositol or PI) in *M. smegmatis* wild-type, Δ*atpD*, and Δ*atpD*^*C*^ strains. In both panels **(E,F)**, the transcript level in each case is compared with that of *M. smegmatis* WT, which is taken as 1. In panels **(B,E,F)**, data represent an average of at least three independent experiments with standard deviation shown as error bars. ^∗^*P*-value < 0.05; ^∗∗^*P*-value < 0.01; ^∗∗∗^*P*-value < 0.005. Panel **(A)** is created with www.BioRender.com.

Since in MsmΔ*atpD*, an upregulation in WhiB3 suggests dissipation of reductive stress *via* lipid biosynthesis, at this juncture, an analysis of the lipid content of the cell becomes extremely important. Cell membrane lipids are known to play a crucial role in the membrane dynamics during growth and stress (Stallings and Glickman, 2010; Queiroz and Riley, 2017). Mycobacterial cell membrane consists of a plethora of lipids, which function as the structural components and storage bodies for the cell (Queiroz and Riley, 2017). A general pathway for the biosynthesis of these lipids in mycobacteria is depicted in Figure 3A. Major structural and storage mycobacterial lipids include phospholipids such as the cardiolipin (CL), phosphatidylethanolamine (PE), phosphatidylinositol (PI), glycosylated PIs, phosphatidylserine (PS), phosphatidylglycerol (PG; a minor species in mycobacteria), and triacylglycerol (TAG), which is a neutral storage lipid for the cell (Crellin et al., 2013). Furthermore, out of the major phospholipids, CL accounts for the maximum amount (37%) of the total phospholipids in mycobacterial plasma membrane followed by PE (32%) and PI/PIMs (28%) (Crellin et al., 2013). We, therefore, first determined the levels of CL in all the three strains. CL is known to form aggregates within the membrane and is determined using Nonyl Acridine orange (NAO) fluorescent dye, which specifically binds to it (Mileykovskaya et al., 2001). Interestingly, the Δ*atpD* bacterium shows reduced fluorescence as compared to that of the wild-type and the complemented strain (Figure 3B), which is further corroborated well by the FACS analysis (Figure 3C). To gain further insight into the reduction in the cardiolipin levels in knock-out strain, we analyzed the mRNA expression level of plasma membrane biogenesis genes (depicted in Figure 3D) by reverse transcription-quantitative PCR (RT-qPCR). We observe several folds reduction in the expression of all the plasma membrane biogenesis genes (*pgsA1*, *pgsA2*, which is a putative cardiolipin synthase gene, and *pgsA3*) in the knock-out strain as compared to the wild-type and the complemented strain (Figure 3E). Overall, this significantly reduced fluorescence and the gene expression levels in the knock-out strain are together suggestive of low amounts of cardiolipin (CL) content in the Δ*atpD* strain as compared to the wild-type and the complemented strain. This also manifests a compromised cell envelope in *M. smegmatis* Δ*atpD* as shown in our previous study (Patil and Jain, 2019). Next we examined the levels of other major phospholipids in the cell such as Phosphatidylethanolamine or PE (*MSMEG_6851*), Phosphatidylserine or PS (*MSMEG_0860*), and Phosphatidylinositol or PI (*MSMEG_2933*) by RT-PCR as well as by monitoring the incorporation of ^14^C oleic acid into phospholipids of all the three strains. Interestingly, our data show that the levels of these phospholipids remain almost unchanged in both wild-type and mutant strains (Figure 3F and Supplementary Figure 3). Taken together, these results suggest that the mycobacterial cell under energy-compromised state might depend on some other lipids in order to maintain survival.

**FIGURE 4:**
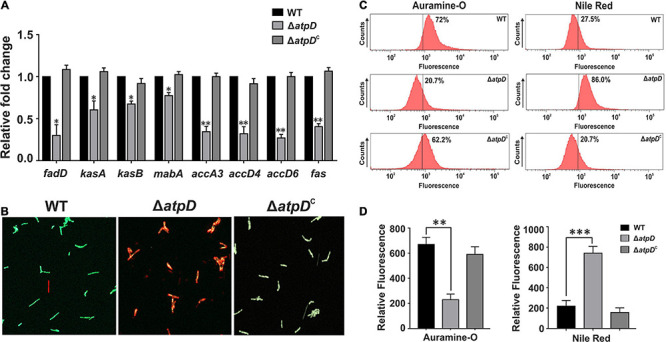
*Mycobacterium smegmatis* shows reduced mycolic acid levels leading to loss of acid-fastness under energy-compromised state. Panel **(A)** shows the relative mRNA transcript levels of the genes belonging to the FAS-II and FAS-I lipid biosynthesis pathway genes such as *fabD* (MSMEG_4325), *kasA* (MSMEG_4327), *kasB* (MSMEG_4328), *mabA* (MSMEG_3150), *accA3* (acetyl-CoA carboxylase; MSMEG_1807), *accD4* (MSMEG_6391), *accD6* (MSMEG_4329), and *fas* (MSMEG_4757) in *M. smegmatis* wild-type (WT), *atpD* knock-out (Δ*atpD*), and *atpD*-complemented (Δ*atpD*^*C*^) strains. The transcript level in each case is compared with that of *M. smegmatis* wild-type, which is taken as 1. Panel **(B)** shows the fluorescence microscopy images for the *M. smegmatis* WT, Δ*atpD*, and Δ*atpD*^*C*^ strains after staining the cells with Auramine-O and Nile red. Panel **(C)** shows the flow cytometric analysis of all the three strains after staining the cells with Auramine-O and Nile red dye, as mentioned. The percentage value represents the fraction of cells that are positive for that fluorescence. Flow cytometry experiments were repeated at least thrice. Panel **(D)** shows the quantification by measuring the fluorescence of Auramine-O and Nile red dyes as mentioned on fluorescence plate reader for *M. smegmatis* WT, Δ*atpD*, and Δ*atpD*^*C*^ strains. In all the panels, the experiments were repeated at least thrice. In both panels **(B,C)**, only one representative image is shown. In panels **(A,D)**, error bars represent standard deviation. ^∗^*P*-value < 0.05; ^∗∗^*P*-value < 0.01; ^∗∗∗^*P*-value < 0.005.

Since mycolic acid acts as the major component of the mycobacterial cell wall, we next asked if the levels of mycolic acid are affected in *M. smegmatis* Δ*atpD*. To investigate this, we first examined the transcription level of the genes belonging to the FAS-I and FAS-II pathway involved in the biosynthesis of mycolic acid. Surprisingly, several-fold downregulation in the expression level of the genes belonging to the FAS-II and FAS-I pathway such as *fabD* (MSMEG_4325), *kasA* (MSMEG_4327), *kasB* (MSMEG_4328), *mabA* (MSMEG_3150), *accA3* (acetyl-CoA carboxylase; MSMEG_1807), *accD4* (MSMEG_6391), *accD6* (MSMEG_4329), and *fas* (MSMEG_4757) is observed in the *atpD* knock-out strain as compared to the wild-type and the complemented strain (Figure 4A). The observed downregulation in the FAS-I and FAS-II pathway genes suggests an alteration in mycolic acid levels (Shi et al., 2010) in the mutant as compared to the wild-type strain. Taken together, this indicates that the deletion of *atpD* gene negatively influences the *de novo* lipid biosynthesis. Further it is well known that alteration in the mycolic acid biosynthesis within the cell leads to loss of acid-fastness in mycobacteria (Daniel et al., 2011, 2016). Therefore, we investigated whether such phenotype is also shown by Δ*atpD*. Therefore, the cells were stained with Auramine-O (acid-fast staining green fluorescent dye), which stains mycolic acids present in the cell envelope of mycobacteria and has been widely used to determine the acid-fastness property of the cells. Here, the *atpD* knock-out strain shows reduced Auramine-O fluorescence indicating loss of acid fastness (Figure 4B). On the other hand, both the wild-type and the complemented bacteria (Δ*atpD*^*C*^) are acid-fast positive as judged by the retention of Auramine-O fluorescence (Figure 4B). Additionally, the mutant shows increased Nile red fluorescence, which is indicative of neutral lipid accumulation, as compared to the wild-type and the complemented strain (Figure 4B). Further, in order to obtain a quantitative estimation, we analyzed Auramine-O and Nile red fluorescence *via* FACS analysis and also measured the fluorescence on a SpectraMax multimode plate reader. Our results clearly show a significant reduction in the Auramine-O fluorescence and a drastic increase in the Nile red fluorescence in Δ*atpD* strain as compared to the wild-type and the complemented strain (Figures 4C,D). It is worth mentioning here that loss of acid-fastness is a feature reported for dormant mycobacteria (Daniel et al., 2011, 2016). Altogether, this remarkable alteration in the cardiolipin, other phospholipids, and mycolic acid levels in Δ*atpD* strongly points toward a significant rerouting of the lipid metabolism to other form in the mutant strain under energy-compromised state.

### Upregulation of WhiB3 Promotes Physiological and Metabolic Adaptations by Increasing TAG Accumulation in *M. smegmatis* Under Energy-Compromised State

**FIGURE 5:**
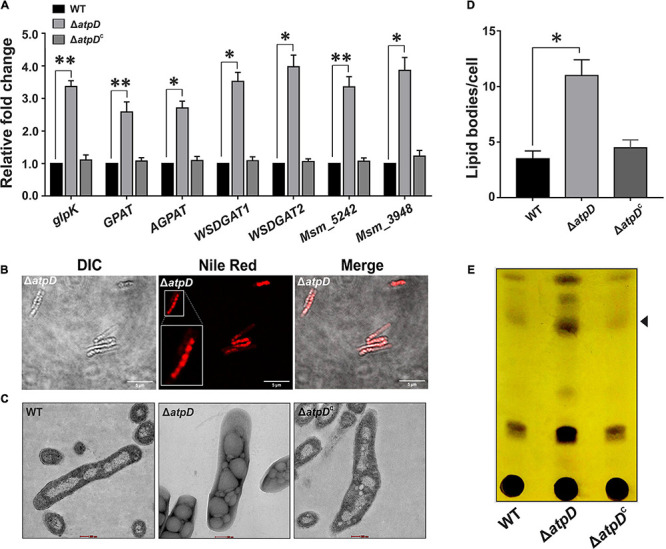
Increased TAG accumulation in *M. smegmatis* under energy-compromised state. Panel **(A)** shows the relative mRNA transcript levels of genes belonging to Kennedy pathway such as glycerol kinase (*glpK)*, glycerol-3 phosphate acyl transferase (*GPAT*), acylglycerol phosphate acyltransferase (*AGPAT*), two wax ester synthase/acyl-CoA: diacylglycerol acyltransferase (*WSDGAT*) and the TAG synthase genes (*MSMEG_3948* and *MSMEG_5242*; these are homologues of *tgs1* in *M. tuberculosis*) in *M. smegmatis* wild-type (WT), *atpD* knock-out (Δ*atpD*), and *atpD*-complemented (Δ*atpD*^*C*^) strains. The transcript level in each case is compared with that of *M. smegmatis* WT, which is taken as 1. Panel **(B)** shows the confocal microscopy images after staining the cells with the Nile red dye. A single zoomed-in image in the Nile red panel is presented as inset for clarity. Panel **(C)** shows the transmission electron microscopy (TEM)-based ultrastructural analysis of all the three strains for the presence of lipid bodies (LBs). The scale bar in each image corresponds to 200 nm. Panel **(D)** shows the quantification of the LBs (*n* = 23) for the images obtained in panel **(C)**. Panel **(E)** depicts the TLC image presenting the TAG accumulation in *M. smegmatis* WT, *atpD* knock out (Δ*atpD*), and Δ*atpD*^*C*^ strains. Spot corresponding to the TAG is marked with an arrowhead. In panels **(A,D)**, data show an average of at least three independent experiments with error bars representing standard deviation. ^∗^*P*-value < 0.05; ^∗∗^*P*-value < 0.01. In panels **(B,C,E)**, only one representative image is shown.

In *M. tuberculosis*, WhiB3 is suggested to modulate the differential production of cell wall associated and storage lipids *via* redox-dependent switching mechanism (Singh et al., 2009); our data also suggest a significant rerouting to some other form of lipid in MsmΔ*atpD*. Since TAG is the major storage lipid in mycobacteria (Daniel et al., 2011), we next explored the genetic basis of this phenomenon in Δ*atpD*, and examined the differential expression of the genes involved in TAG biosynthesis pathway, which is also known as Kennedy pathway in *M. smegmatis* (Maarsingh and Haydel, 2018). Interestingly, we find that the transcription profile of the genes coding for the enzymes catalyzing the TAG biosynthesis such as glycerol kinase (*glpK*, MSMEG_6759), glycerol-3-phosphate acyl transferase (GPAT, MSMEG_4703), acylglycerol phosphate acyltransferase (AGPAT, MSMEG_4284), and the two wax ester synthase/acyl-CoA:diacylglycerol acyltransferase (WSDGAT, MSMEG_1882, and MSMEG_4705), which catalyzes the final step of TAG biosynthesis, is upregulated in the Δ*atpD* mutant as compared to the wild-type and the complemented strain (Figure 5A). In *M. tuberculosis*, *tgs1* is known to be the major TAG synthase gene (Daniel et al., 2004), and we found the two homologs of *tgs1* genes in *M. smegmatis*, MSMEG_5242 and MSMEG_3948, which bear significant similarity (69%) with the *M. tuberculosis tgs1* (Supplementary Figure 4). Interestingly, the transcript levels of these genes are also found to be higher (Figure 5A), which strongly suggests that this phenomenon may be conserved in *M. tuberculosis* as well. We additionally noticed that in *M. tuberculosis*, *tgs1* gene is under direct control of *dosS/dosR* operon, and is highly overexpressed under stress conditions such as hypoxia, acidic environment, and under dormancy conditions (Daniel et al., 2011). Our previous report also showed an overexpression of dormancy regulon genes *dosS* and *dosR* that are the known mediators of *tgs1*-dependent TAG accumulation (Patil and Jain, 2019). Thus, an overexpression of TAG synthase genes in *M. smegmatis* Δ*atpD* (under an energy-compromised state) points toward the dormancy related metabolic state in the cell.

The upregulation of the Kennedy pathway in the Δ*atpD* mutant strongly supports our hypothesis that the mutant retains TAG as the major storage lipid. To further confirm this finding, the mutant cells were stained with Nile red dye that specifically stains neutral lipids within the cell. Our confocal microscopy data clearly shows the presence of large number of brightly stained lipid bodies (LBs) in the knockout strain (Figure 5B). Transmission electron microscopy (TEM) analysis of the clinical strains of *M. tuberculosis* carried out by Vijay and coworkers clearly demonstrated a distinct accumulation of LBs containing TAG (Vijay et al., 2017). Therefore, in order to visualize the LBs in *M. smegmatis*, we performed its ultrastructural analysis using TEM. Our data clearly show the presence of large amount of LBs in the Δ*atpD* bacterium as compared to the wild-type and the complemented strain (Figures 5C,D). We additionally examined the levels of TAG in the mycobacterial cells using thin layer chromatography (TLC) wherein the knock-out strain shows a clear accumulation of TAG as an intense band compared to that obtained in the wild-type and the complemented strain (Figure 5E). Furthermore, in order to confirm that the free fatty acids (FFAs) incorporated within the knockout cells are mainly stored as TAG rather that simple FFA or phospholipids (McCarthy, 1971; Weir et al., 1972; Nakagawa et al., 1976; Barksdale and Kim, 1977), we used ^14^C oleic acid as a radiolabelled substrate and monitored the incorporation of ^14^C oleic acid as it acts as the major component of TAG. All the three bacteria *viz*. wild-type, Δ*atpD*, and Δ*atpD*^*C*^ were grown in the presence ^14^C-Oleic acid, and the radiolabeled lipids were extracted and analyzed by silica-based TLC. We observe that in the knock-out strain, the incorporation of ^14^C-Oleic acid in TAG is significantly higher as compared to wild-type and complemented strain (Supplementary Figure 5). This further strengthens our observation that the *atpD* mutant retains TAG as the storage lipid.

### TAG Hydrolysis Is Mediated by Intracellular Lipase Enzymes and the Consumption Can Be Blocked by Lipase Inhibitor

**FIGURE 6:**
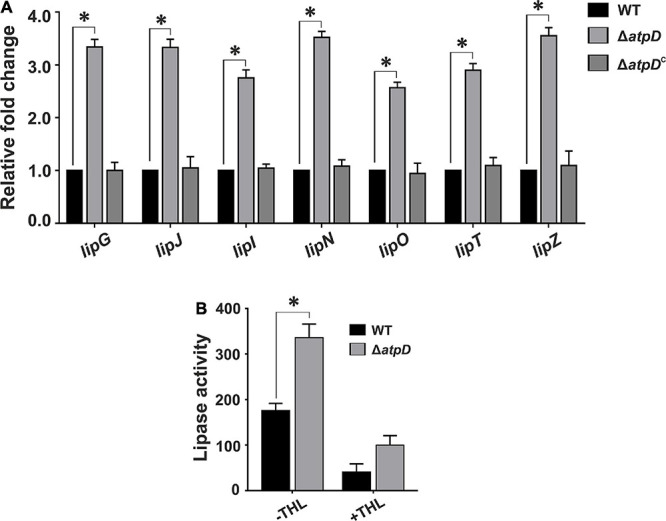
*Mycobacterium smegmatis* Δ*atpD* shows higher lipase gene expression coupled with an increased lipase activity. Panel **(A)** shows the relative mRNA transcript levels of the lipase genes (*lipG, lipJ, lipI, lipN, lipO, lipT*, and *lipZ*) in *M. smegmatis* wild-type (WT), *atpD* knock out (Δ*atpD*), and *atpD*-complemented (Δ*atpD*^*C*^) strains. The transcript level in each case is compared with that of *M. smegmatis* wild-type, which is taken as 1. Panel **(B)** shows the lipase activity in *M. smegmatis* WT and its *atpD* knock-out (Δ*atpD*) variant measured in the absence (–THL) and in the presence (+THL) of lipase inhibitor tetrahydrolipstatin (THL). In both the panels, data represent an average of at least three independent experiments with error bars depicting standard deviation. ^∗^*P*-value < 0.05.

During dormancy, *M. tuberculosis* is known to utilize accumulated TAGs as energy source (Daniel et al., 2011), wherein FFAs are released *via* lipase-mediated hydrolysis, which further participate in β-oxidation. Therefore, in order to understand if *M. smegmatis* Δ*atpD* preferentially utilizes TAG as an energy source, we examined the expression profile of various intracellular lipase genes in *M. smegmatis*. Interestingly, we observe at least two- to threefold higher expression of several lipase (*lip*) genes such as *lipG*, *lipJ*, *lipI*, *lipN*, *lipO*, *lipT*, and *lipZ* in *M. smegmatis* Δ*atpD* as compared to the wild-type and the complemented bacterium (Figure 6A), indicating the possibility of enhanced TAG hydrolysis. In *M. tuberculosis*, lipase activity has been suggested to be regulated at post-translational level (Ravindran et al., 2014). Hence to confirm that enhanced lipase gene expression also results in enhanced lipase production, we measured the lipase activity in *M. smegmatis* Δ*atpD* in the presence and absence of a pathway-independent lipase inhibitor, tetrahydrolipstatin (THL). Lipase inhibitors are well-known to interfere with the lipid metabolism by impairing the activity of cellular lipolytic enzymes (Lass et al., 2011; Zechner et al., 2012). These lipid inhibitors are used to detect the molecular basis of TAG accumulation and its metabolism (Lass et al., 2011; Zechner et al., 2012). Our data show that in *M. smegmatis* Δ*atpD*, higher lipase expression coincides well with higher lipase activity that is reduced in the presence of inhibitor (Figure 6B). Overall our results suggest the importance of both accumulation and possible hydrolysis of TAGs for the energy production under an energy-compromised state.

**FIGURE 7:**
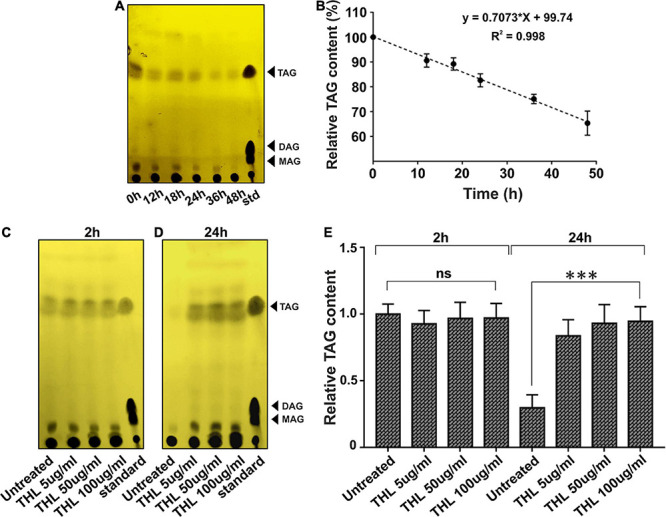
Intracellular lipolytic enzymes mediate TAG hydrolysis in *M. smegmatis* Δ*atpD*. Panel **(A)** shows the TLC image for the triacylglycerol (TAG) accumulation and its disappearance in *M. smegmatis* Δ*atpD* upon starvation with time (in hours). TAG mobility was compared with standard (std). Spot corresponding to the TAG, diacylglycerol (DAG), and monoacylglycerol (MAG) is marked with an arrowhead. Panel **(B)** shows the relative quantification of TAG by densitometric analysis of the TLC with time (in hours). Here, 100% corresponds to the 0 h time point. Panels **(C,D)** depict the TLC image showing the TAG accumulation and disappearance after the treatment of cells with a lipase inhibitor tetrahydrolipstatin (THL) at 2 h **(C)** and 24 h **(D)** time period. “Untreated” represents cells without THL treatment. “standard” is similar to “std” in panel **(A)**. Panel **(E)** shows the relative TAG content as assessed by densitometric analysis of the TLC image, obtained before and after treating the cells with the increasing concentration of THL. The experiments were repeated at least thrice. In panels **(A,C,D)**, only one representative image is shown. In panels **(B,E)**, error bars represent standard deviation. ^∗∗∗^*P*-value < 0.005; “ns,” not significant.

In order to further confirm if Δ*atpD* preferentially utilizes TAG as an energy source, we monitored the TAG consumption in Δ*atpD*. We first grew the mutant strain for 24 h to allow for the accumulation of TAG. The cells were then transferred to a fresh medium without any carbon source and the TAG utilization was monitored for 48 h. Our results clearly show a linear decrease in the relative TAG content as a function of time, which suggests TAG hydrolysis in the mutant strain (Figures 7A,B). To further confirm that mycobacterial lipases hydrolyze TAG, the mutant bacteria were treated with an increasing concentration of THL for 2 h. Thereafter, the cells were washed and resuspended in fresh medium containing increasing concentrations of THL (5, 50, and 100 μg/ml) and were further incubated for 24 h. The samples were collected and TAG content was analyzed by TLC. The data show no significant difference in the TAG levels at 2 h time point in the presence or absence of the inhibitor (Figure 7C). However, interestingly, after 24 h of incubation, while the untreated cells show a significant disappearance of TAG, the cells exposed to increasing concentration of THL show the presence of TAG (Figures 7D,E). Taken together, our data suggest that *M. smegmatis* utilizes stored LBs containing TAG during energy depletion state by means of hydrolyzing neutral lipids. We wish to add here that the TAG consumption in Δ*atpD* also depends upon the expression of endogenous lipase enzymes, which further leads to the release of FFA. These FFAs are then utilized by the cell fueling the lipid oxidation pathways such as β-oxidation supporting the cell survival. The end product of the β-oxidation pathway is acetyl-CoA, which is directly channeled into the glyoxylate shunt to acquire cellular energy, thereby utilizing fatty acid as the major carbon source (Serafini et al., 2019). Indeed an upregulation of the β-oxidation pathway and glyoxylate shunt in *M. smegmatis* Δ*atpD*, as shown previously (Patil and Jain, 2019), corroborates well with these observations.

### Bedaquiline-Treated Wild-Type *M. smegmatis* Also Shows Increased WhiB3 Expression Coupled With TAG Accumulation and Lipase Gene Upregulation

Bedaquiline (BDQ) is known as the first-in-class ATP synthase inhibitor, having an extremely high selectivity for mycobacterial ATP synthase (Lakshmanan and Xavier, 2013). Upon BDQ exposure, *M. tuberculosis* has been shown to undergo significant metabolic remodeling along with reduced ATP/ADP levels (Koul et al., 2014; Akela and Kumar, 2021). BDQ inhibits bacterium’s ability to utilize OXPHOS and forces the cell to rely upon substrate-level phosphorylation, thereby continuing with ATP generation (Smith et al., 2020). Although an adequate amount of energy is provided by substrate-level phosphorylation for the cell survival, mycobacteria majorly depend upon OXPHOS to sustain their basic energy requirements (Foo et al., 2020). Hence, BDQ treatment in mycobacteria is thought to disturb the cellular environment forcing the cell to reroute its metabolism to overcome the stressful condition.

**FIGURE 8:**
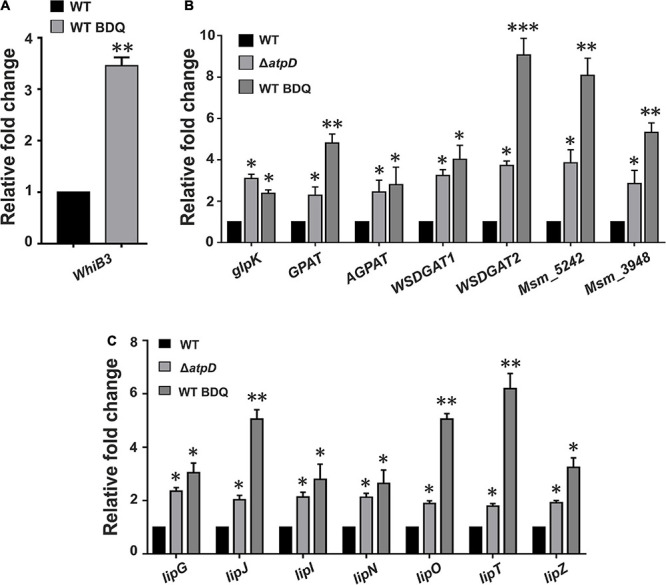
Bedaquiline-treated wild-type *M. smegmatis* shows increased WhiB3 expression coupled with TAG accumulation and lipase gene upregulation. Panel **(A)** shows the mRNA transcript level of redox sensor WhiB3 in wild-type *M. smegmatis* cell either without (WT) or with bedaquiline (BDQ)-treatment (WT BDQ); for comparison with the *M. smegmatis atpD* knock-out strain, refer to Figure 2A. Panel **(B)** shows the relative mRNA transcript levels of genes belonging to Kennedy pathway such as glycerol kinase (*glpK*), glycerol-3 phosphate acyl transferase (*GPAT*), acylglycerol phosphate acyltransferase (*AGPAT*), two wax ester synthase/acyl-CoA: diacylglycerol acyltransferase (*WSDGAT*) and the TAG synthase genes (*MSMEG_3948* and *MSMEG_5242*; these are homologues of *tgs1* in *M. tuberculosis*) in *M. smegmatis* WT, its *atpD* knock-out variant (Δ*atpD*), and the bedaquiline (BDQ)-treated wild-type *M. smegmatis* (WT BDQ). Panel **(C)** shows the relative mRNA transcript levels of the lipase genes (*lipG, lipJ, lipI, lipN, lipO, lipT*, and *lipZ*) in *M. smegmatis* WT, *atpD* knock-out (Δ*atpD*), and the bedaquiline (BDQ)-treated wild-type *M. smegmatis* (WT BDQ). In all of the panels, the mRNA transcript in the case of untreated WT is taken as 1 for comparison. In each panel, the data represent an average of at least three independent experiments. Error bars represent standard deviation. ^∗^*P*-value < 0.05; ^∗∗^*P*-value < 0.01; ^∗∗∗^*P*-value < 0.005.

An earlier work suggested that bactericidal antibiotics induce ROS formation in *M. tuberculosis* leading to alterations in cellular redox state (Dwyer et al., 2014). Additionally, BDQ in combination with other antibiotics such as clofazimine and telacebec is very well known to induce oxidative stress and ROS formation with increased cellular reductive stress in *M. tuberculosis* (Lamprecht et al., 2016). Furthermore, since *M. smegmatis* Δ*atpD* balances the cellular reductive stress *via* WhiB3 overexpression, we asked if this mechanism persists in *M. smegmatis* upon BDQ treatment also. Hence we determined the WhiB3 expression under BDQ-treated condition. Interestingly, BDQ-treated wild-type *M. smegmatis* shows ∼3.5-fold upregulation of the WhiB3 expression (Figure 8A). Moreover, since our previous report also suggests that *M. smegmatis* Δ*atpD* strain largely mimics BDQ-treated wild-type *M. smegmatis* (Patil and Jain, 2019), at this juncture, we asked if WhiB3 overexpression in BDQ-treated wild-type *M. smegmatis* also accumulates TAG, thereby dissipating the reductive stress and utilizing TAG as an energy source for the cell survival. Our results clearly show that BDQ-treated wild-type *M. smegmatis* also overexpresses TAG biosynthesis pathway genes (*glpK*, GPAT, AGPAT, WSDGAT1, WSDGAT2, MSMEG_3948, and MSMEG_5242) similar to the untreated Δ*atpD* strain (Figure 8B). Additionally, BDQ treatment also results in overexpression of lipase genes (Figure 8C), suggesting the possibility of increased TAG hydrolysis upon treatment with BDQ. These results strongly indicate that upon BDQ treatment, wild-type *M. smegmatis* stores as well as consumes TAG to meet its energy requirements, which mimics the Δ*atpD* strain in the absence of BDQ. This observation has a significant impact, since it is widely accepted that *M. tuberculosis* switches from carbohydrate to host fatty acids in the phagosome, and balances the reductive stress (Singh et al., 2009). Taken together, our data indicate a critical role of redox sensitive WhiB3 in regulating crucial aspects in mycobacterial physiology and survival during BDQ-treatment.

## Discussion

*Mycobacterium tuberculosis* exhibits a remarkable metabolic flexibility and robust ETC to survive under various stress conditions (Iqbal et al., 2018). Thus OXPHOS in mycobacteria is a tightly regulated process. However, the restricted respiration due to O_2_ depletion or by inhibition of ETC complexes induces changes in the mycobacterial respiratory machinery making the electron carriers to be more reduced thereby leading to alteration in the redox homeostasis (Bhat et al., 2012). Bacteria utilize a large number of dehydrogenases and redox sensors in order to dissipate reductive stress caused by an excessive accumulation of NADH and to replenish the cellular NAD^+^ levels (Bhat et al., 2012). For example, the Rex repressors in the Gram-positive bacteria are known to regulate the gene expression changes in response to altered NADH/NAD^+^ ratios (Gyan et al., 2006). The Fnr regulators also sense the intracellular O_2_ levels *via* (4Fe-4S) cluster and thereby regulate the cellular response (Kiley and Beinert, 1998). Similarly, the RegB histidine kinase (HK) of the *Rhodobacter capsulatus* two component system (TCS) RegBA, and the AcrB HK of *E*scherichia *coli* TCS AcrBA were also suggested to be upregulated under anaerobic conditions (Bekker et al., 2010). However, homologs of none of these genes are present in *M. smegmatis*.

The DosSR TCS in mycobacteria consists of two heme-based HK, DosS, and DosT, that directly sense the intracellular concentration of O_2_ in the cell, and relays the signal to a response regulator DosR, which upregulates the target responsive dormancy regulon genes under reduced oxygen conditions (Kim et al., 2010; Tan et al., 2010). Some major dormancy regulon genes upregulated during reduced oxygen include nitrate reductase (*narK2* and *narX*), fumarate reductase (Frd, *frdABCD*), ferredoxin (*fdxA*), DNA repair genes (*nrdZ*) and TAG biosynthesis genes (*tgs1*) (Tan et al., 2010). In addition, upon exposure to variety of stress (reduced O_2_, NO, and CO), the DosSR TCS gets activated leading to an increase in the cellular NADH pools, thus playing an important role during reductive stress in mycobacteria (Kumar et al., 2007; Honaker et al., 2010). Another major redox regulator in mycobacteria is WhiB3 that senses the intracellular redox state, and helps in the dissipation of the reductive stress generated due to accumulated NADH cofactor *via* lipid biosynthesis (Singh et al., 2009). The metabolic shift induced by WhiB3 regulates the production of methyl branched polyketides (PAT/DAT, SL-1, and PDIM) and TAG (Singh et al., 2009). Synthesis of these lipids by WhiB3 consumes NADH, replenishing the cellular NAD^+^ levels and relieving the cell from reductive stress (Singh et al., 2009). Interestingly, the TAG synthase *tgs1* gene is under the direct control of DosSR operon (Kumari et al., 2017). Lipid inclusions are highly prevalent amongst various prokaryotic organisms such as *Rhodococcus*, *Streptomyces*, *Nocardia*, and *Pseudomonas aeruginosa* and in eukaryotic organisms like *Saccharomyces cerevisiae*. In mycobacteria, TAG accumulation has recently received renewed interest. The W-Beijing lineage of *M. tuberculosis* also shows an upregulation of DosR/S operon with an overproduction of TAG levels. LB containing TAG are the most prominent storage fuel in mycobacteria that not only are essential for prolonged survival, infection, dormancy, and persistence, but are also required for the reactivation of growth following dormancy (Daniel et al., 2011). Similarly, the sputum analysis of pulmonary TB patients and MDR strains of *M. tuberculosis* also show the presence of TAG containing LB, suggesting importance of TAG accumulation in TB pathogenesis and infection (Vijay et al., 2017). Hence TAG acts as a major redox sink to dissipate reductive stress in the cell.

**FIGURE 9:**
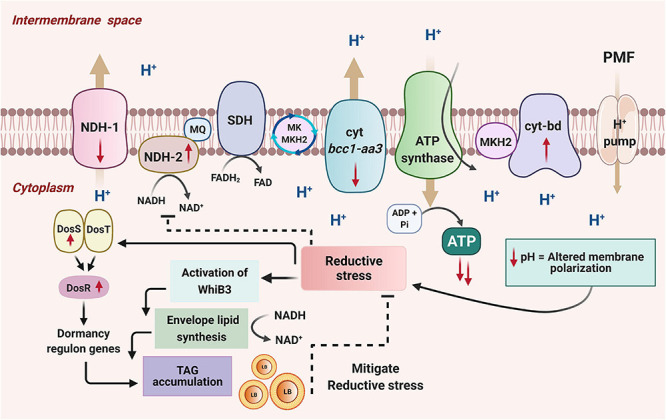
A model depicting the adaptation of *M. smegmatis* under energy-compromised state. Disruption of one copy of *atpD* in *M. smegmatis* drives the transcriptional changes in the mycobacterial ETC. The energy efficient complexes (NDH1 and cyt *bc_1_-aa_3_*) are down-regulated while the energy less efficient complexes (NDH2 and cyt *bd* oxidase) are upregulated as shown by the direction of the red arrows. This leads to the remodeling of the respiration chain, and, as a result, the protons get trapped in the cytoplasmic region as they are now unable to translocate to the periplasmic region, resulting in the decrease in cellular pH. This causes a change in the membrane polarization, and leads to the reductive stress in the cell. This activates a redox sensor, WhiB3, which dissipates the reductive stress by promoting biosynthesis of cell wall associated lipids and by increasing TAG accumulation. WhiB3-mediated lipid biosynthesis helps in the regeneration of NAD^+^ from NADH, which helps in mitigating the reductive stress. The accumulated TAG forms lipid bodies (LB). Mycobacteria also possess another sensing mechanism DosSRT dormancy regulon, which detects changes in cellular redox state through the heme proteins of its two sensor kinases, DosS and DosT. This transfers the signal to the response regulator DosR, which mediates the upregulation of dormancy regulon genes. An essential gene of this regulon that gets activated during stress is the *tgs1*, which is the major TAG regulator gene, mediating production and accumulation of lipid bodies (LB) containing TAG, thereby dissipating the reducing equivalents and facilitating mycobacterial survival under energy-compromised state. Hence these combined effects of WhiB3 and Dos regulon mediated signaling pathways function to overcome the energy demand and maintain the redox homeostasis in *M. smegmatis* under energy-compromised state. Figure is created with www.BioRender.com.

In the present work, we report a critical outcome of the deletion of one of the two copies of *atpD* gene in *M. smegmatis*. We have previously shown that deletion of one copy of *atpD* gene in *M. smegmatis* renders the cell in an energy-compromised state (Patil and Jain, 2019). Here we investigated the adaptive mechanisms undertaken by the bacterium during such condition. We show that *M. smegmatis* under energy compromised state exhibits restricted respiration by downregulating the energy efficient ETC complexes *viz.* NDH1 and cyt *bc1-aa3* complex and upregulating energetically less efficient ETC complexes *viz.* NDH2 and cyt-*bd* oxidase complex. This broad shift in the respiratory machinery results in the trapping of protons in the cytoplasmic region, resulting in the acidification of cellular pH. This change alters the membrane polarization as seen by an increase in DiBAC_4_(3) fluorescence in the knockout cells as compared to the wild-type. Moreover, a higher NADH/NAD^+^ ratio and an increased ROS levels induces an altered redox state. Finally, in order to dissipate these reducing equivalents and to maintain intracellular redox balance, WhiB3, a major redox sensor, is upregulated and it maintains the redox homeostasis *via* modulating the biosynthesis of cell wall-associated lipids and TAG resulting in an accumulation of LBs. Our results show that Δ*atpD* bacterium tends to accumulate more LBs than the wild-type bacterium, indicating that TAG accumulation is a prominent metabolic adaptation in Δ*atpD* under energy-compromised state. Additionally, by using ^14^C oleic acid, we further show that the FFAs incorporated within the knockout cells are mainly stored as TAG rather that simple FFA or phospholipids. Furthermore, the *atpD* bacterium also shows loss of acid fastness and significantly down-regulated cardiolipin biosynthesis gene expression which is most likely the result of an upregulated TAG biosynthesis pathway in Δ*atpD*. With an upregulation of TAG pathway, we also observed a concomitant overexpression of intracellular lipases genes, suggesting that the accumulated TAG is hydrolyzed for its utilization. Furthermore, an overproduction of lipases along with the previously reported enhanced β-oxidation (Patil and Jain, 2019) together indicates that the stored lipids are used for energy production (Figure 9). This likely happens because the Δ*atpD* bacterium is unable to meet its energy requirements by OXPHOS due to lesser availability of functional ATP synthase. It is worth adding here that the catabolism of the fatty acids under such energy-depleted state leads to the production of acetyl CoA, which is then metabolized *via* glyoxylate shunt in order to avoid carbon loss *via* TCA oxidation. Hence the lipids are acted upon by the lipases to release FFAs, which are then metabolized *via* β-oxidation generating a pool of acetyl CoA, which thereafter enters the glyoxylate cycle producing energy for cell survival (Patil and Jain, 2019).

Interestingly, wild-type *M. smegmatis* upon BDQ treatment also shows an upregulation in WhiB3 expression, suggesting an important role of WhiB3 mediated redox sensing in mycobacterial virulence under BDQ-treated conditions. Additionally, a predominance of TAG biosynthesis coupled with an increased lipase production is also observed similar to Δ*atpD*. This phenomenon appears to be conserved in *M. tuberculosis*; indeed, proteomic analysis of *M. tuberculosis* treated with BDQ showed an upregulation of TAG biosynthesis gene products at 6 and 24 h time (Koul et al., 2014). This strongly indicates that the survival response of bacterium to BDQ treatment is identical to what we observe upon creating an energy-compromised state by disrupting ATP synthase machinery.

The observed physiological changes presented in this manuscript depict a significant outcome of the metabolic dependency of mycobacteria on fatty acids as an energy source under energy-deprived state and a highly evolved mechanism of dissipating reducing equivalents in the cell *via* TAG metabolism. We thus conclude that mycobacteria exhibit a robust and flexible system that allows rerouting of its central metabolic pathways under an energy-compromised state. Further, since TAG accumulation provides metabolic energy to the dormant cell, we believe that our work will also contributes toward the exploration of newer possible drug targets in eliminating the dormant and persistent pathogenic mycobacteria.

### Experimental Procedures

#### Bacterial Strains, Plasmids, Reagents, Media, and Growth Conditions

Wild-type *M. smegmatis* mc^2^155, *atpD* knock-out (Δ*atpD*), and the complemented strain (Δ*atpD*^*C*^) were grown in Middlebrook 7H9 (MB7H9 broth; Difco) containing 2% glucose and 0.05% tyloxapol surfactant (Sigma-Aldrich, MO, United States) as indicated, at 37°C with constant shaking at 200 rpm. *M. smegmatis*Δ*atpD* and Δ*atpD*^*C*^ were taken from the laboratory stock (Patil and Jain, 2019). The cultures were supplemented with 50 μg ml^–1^ kanamycin (Sigma) or 100 μg ml^–1^ hygromycin (MP Biomedical), wherever required. Protein expression was induced by the addition of 2% acetamide (wherever required).

#### Lipid Extraction and Analysis by Thin Layer Chromatography

Triacylglycerol was extracted following the chloroform:methanol (2:1 v/v) method. Briefly, the bacteria were grown in MB7H9 medium supplemented with 2% glucose and 0.05% tyloxapol till 24 h. The cells were then lyophilized and 300 mg of cells in each case were further used for TAG extraction by chloroform:methanol (2:1 v/v). The lower organic layer was collected, and the lipids were dried and re-suspended in chloroform:methanol (1:1 v/v). Lipids were further separated by TLC using hexane:diethylether:formic acid (40:10:1 v/v) as the mobile phase. The plates were allowed to dry, and stained using 5% phosphomolybdic acid in 95% ethanol followed by charring at 140°C for 5 min.

For TAG hydrolysis experiments, bacteria were grown in MB7H9 medium supplemented with 2% glucose and 0.05% tyloxapol for 24 h. The cells were then harvested by centrifugation at 5000 rpm for 15 min and washed with 1X-PBS buffer. The cells were further resuspended in the media without any carbon source in order to induce starvation. The cells were then incubated at 37°C with constant shaking at 200 rpm, and samples were collected at specific time points. For lipase inhibition experiments, the cells were pre-incubated with lipase inhibitor THL for 2 h. A portion of these cells was then harvested and washed with 1X-PBS buffer, while the remaining cells were further incubated for 24 h, with appropriate THL concentrations (5, 50, and 100 μg/ml^−1^). Cells were then harvested at specific time points and their lipid content was analyzed by TLC. Densitometric analysis was performed using ImageJ software to determine the relative TAG content in the samples.

### Ultrastructure Analysis by Transmission Electron Microscopy

Transmission electron microscopy was carried out with *M. smegmatis* cells essentially as described previously (Gupta et al., 2015; Patil and Jain, 2019) with some modifications. Briefly, the bacterial cell pellet was obtained from 24 h grown culture. The cells were fixed in 0.15 M sodium cacodylate buffer containing 2% osmium tetraoxide for 2 h at RT. The cells were then washed with cacodylate buffer followed by fixation for 3 h at RT in 0.15 M sodium cacodylate buffer containing 2% tannic acid and 2% glutaraldehyde solution. The cells were then washed again with cacodylate buffer, and re-fixed with 2% osmium tetraoxide overnight. Samples were then washed with Milli-Q water and embedded in 2% molten agarose. The finely cut agar block pieces were then subjected to sequential acetone dehydration at increasing concentrations for 30 min each (30, 50, 70, 90, and 100%), followed by infiltration and embedding in Spurr’s resin. After ultra-sectioning, the samples were stained with uranyl acetate and phosphotungstic acid, imaged on FEI Talos 200S system, equipped with a 200 kV Field Emission Gun, and the LBs were counted manually.

### Auramine-O and Nile Red Microscopy and Fluorescence Measurements

For fluorescence microscopy, the fluorescent acid-fast staining dye Auramine-O was used along with a neutral lipid staining dye Nile red (9-diethylamino-5H-benzo-a-phenoxazine-5-one) using the protocol as described previously (Deb et al., 2009) with some modifications. Briefly, the log phase cultures (OD_600_ ∼0.6) of wild-type *M. smegmatis* mc^2^155, Δ*atpD*, and Δ*atpD*^*C*^ were harvested and washed with 1X-PBST (0.137 M NaCl, 0.0027 M KCl, 0.01 M Na_2_HPO_4_, 0.0018 M KH_2_PO_4_, 0.05% Tween-20). Thereafter, the cultures were evenly spread to make a smear on a glass slide, heat fixed, covered with Auramine-O (10 mg ml^–1^), and incubated for 20 min. The cells were then washed briefly with water and treated with the decolorizer (acid alcohol) for 30 s. Further, the specimen was washed and covered with Nile red solution (10 mg ml^–1^ in ethanol) and incubated for 15 min. The cells were then washed and covered with potassium permanganate solution for 1 min, washed thoroughly with water, air dried, and imaged on using ApoTome Fluorescence Microscope (Carl Zeiss) using an 100× objective lens.

For Nile red confocal microscopy experiments, the Δ*atpD* cells were grown for 24 h. The cells were then harvested and washed with 1X-PBS buffer and further resuspended in it. Nile red dye (10 mg/ml in ethanol) was added to the cell suspension. Cells were further incubated for 30 min in dark at 37°C. The stained cells were then harvested and washed twice with 1X-PBST buffer and finally resuspended in it. A total of 2 μl of bacterial suspension was spotted on 1% agarose pad. Bacteria were then analyzed by capturing the images on Olympus FV3000 confocal microscope with 100× oil-objective.

The flow cytometry based FACS measurements were performed using BD-FACS Aria III equipped with CELL QUEST software (South San Francisco, CA, United States). For measuring the Auramine-O and Nile red fluorescence, log phase cultures of all the strains were harvested, and washed with 1X-PBST. Both Auramine-O and Nile red dyes were added to the samples at a final concentration of 10 mg/ml. Samples were incubated at room temperature in dark for 1 h, washed twice and re-suspended in 1X-PBST buffer. Flow cytometry was performed using FITC and Texas Red filter for Auramine and Nile red, respectively. Alternatively, fluorescence was measured on SpectraMax M5 plate reader with Ex/Em wavelengths set at 438/505 nm for Auramine-O and at 550/640 nm for Nile red.

### Assessment of the Presence of Cardiolipin by Staining With Nonyl Acridine Orange

The flow cytometry based FACS measurements were performed using BD-FACS Aria III equipped with cell quest software (South San Francisco, CA, United States). The total cardiolipin levels were measured using NAO (10-Nonyl Acridine orange). Briefly log phase cultures (OD_600_ ∼0.6) of all the strains were harvested, and washed with 1X-PBST. The cells were then incubated with 10 μM NAO for 30 min at room temperature, washed twice and re-suspended in 1X-PBST buffer. Fluorescence was then measured using FITC filter for flow cytometry, and, alternatively, on Spectramax M5 plate reader with Ex/Em wavelengths set at 495/519 nm.

### Metabolic Incorporation of Radiolabeled Oleic Acid by *Mycobacterium smegmatis*

Wild-type *M. smegmatis* and its variants were grown in MB7H9 medium supplemented with 2% glucose and 0.05% tyloxapol till log phase (OD_600_ ∼0.6) at 37°C with constant shaking at 200 rpm. ^14^C-oleate (50 μCi mol^–1^; 5 μCi per 10 ml of culture; Perkin Elmer, Waltham, MA, United States) was then added in each culture for 30 min. Thereafter, the lipid extraction, TLC, and phosphor imaging procedures were performed as described previously with some modifications (Daniel et al., 2016). Briefly, the cells were harvested by centrifugation at 8000 rpm at 4°C and were washed with 1X-PBST buffer to remove the unincorporated radioactive material. The lipids were then extracted using chloroform:methanol (2:1 v/v). The organic layer was recovered and dried completely. The dried lipids were further dissolved in chloroform:methanol (1:1 v/v) and were subjected to TLC using hexane:ethyl ether:formic acid (40:10:1 v/v) as the mobile phase. For each sample, the amount of radioactivity present was measured on a scintillation counter, and the equal amount of radioactivity was loaded on TLC. The TLC plate was then dried and imaged on a phosphor imager (FLA 9000; GE Healthcare). For phospholipid analysis, ^14^C-oleate (54.3 μCi mmol^–1^; 5 μCi per 10 ml of culture) was added in each culture for 6 h, followed by lipid extraction as stated above. Dried lipids were further dissolved in chloroform:methanol (1:1 v/v). Equal cpm was spotted on TLC, and the lipids were separated using CHCl_3_:CH_3_OH:H_2_O (65:25:4 v/v) as the mobile phase, followed by phosphor imaging procedure as mentioned above.

### Lipase Assay

Lipase activity was assessed using Lipase Assay Kit (Sigma Aldrich, MAK048) following the manufacturer’s instructions with some modifications. Briefly, the log phase cultures were harvested and washed with 1X-PBST. The cells were then resuspended in lipase buffer followed by the addition of lipase inhibitor (tetrahydrolipstatin or THL), wherever required, to a final concentration of 80 μM. The cells were then sonicated and were further processed for lipase assay. Fluorescence was recorded on SpectraMax M5 plate reader with Ex/Em wavelengths set at 529/600 nm.

### RNA Extraction, cDNA Synthesis, and RT-qPCR

Differential gene expression profile was obtained by RT-qPCR for bacteria essentially as described previously (Patil and Jain, 2019). Briefly, the cells were grown till OD_600_ ∼ 0.8. The cells were then harvested, and processed for RNA isolation using RNeasy Mini kit (QIAGEN) by following manufacturer’s instructions. cDNA was synthesized using i-script cDNA synthesis kit (Bio-Rad) as per the manufacturer’s instructions. The obtained cDNA was then used for qPCR experiments, carried out on StepOnePlus real-time PCR system (Applied Biosystems) using iTaq universal SYBR green mix (Bio-Rad) as per the manufacturer’s instructions. *rpoB* gene was used as an internal control. Data were analyzed as per 2^–ΔΔ*Ct*^ method. Primers used for these PCR reactions are listed in Supplementary Table 1.

### Statistical Analysis

Data are represented as arithmetic means in the results that are obtained from at least three independent experiments with standard deviations shown as error bar. Statistical significance was calculated using unpaired *t*-test for data analysis as mentioned in the respective figure legends.

## Data Availability Statement

The original contributions presented in the study are included in the article/Supplementary Material, further inquiries can be directed to the corresponding author/s.

## Author Contributions

VJ and VP designed the research, analyzed the data, and wrote the manuscript. VP performed the research. Both authors contributed to the article and approved the submitted version.

## Conflict of Interest

The authors declare that the research was conducted in the absence of any commercial or financial relationships that could be construed as a potential conflict of interest.

## Publisher’s Note

All claims expressed in this article are solely those of the authors and do not necessarily represent those of their affiliated organizations, or those of the publisher, the editors and the reviewers. Any product that may be evaluated in this article, or claim that may be made by its manufacturer, is not guaranteed or endorsed by the publisher.

## Supplementary Material

Supplementary Figure 1 Pairwise sequence alignment of WhiB3 protein of *M. tuberculosis* and *M. smegmatis*. Pairwise sequence alignment of WhiB3 protein from *M. tuberculosis* (Rv3416) and *MSMEG_1597* from *M. smegmatis* is shown. “^∗^” represents conserved residue.

Supplementary Figure 2 Sequence alignment of polyketide biosynthesis protein of *M. tuberculosis* and *M. smegmatis*. Pairwise sequence alignment of Rv3825c protein (*pks2*) from *M. tuberculosis* and MSMEG_4724 (*pks5*) from *M. smegmatis* is shown. “^∗^” represents conserved residue.

Supplementary Figure 3 Phospholipid analysis by thin layer chromatography. Panel shows the TLC image depicting the phospholipids in *M. smegmatis* wild-type (WT), *atpD* knock out (Δ*atpD*), and *atpD*-complemented (Δ*atpD*^*C*^) strains. Spot corresponding to PE, PI, and PS (top to bottom) is marked with arrowhead. Experiments were repeated at least thrice; only one representative image is shown.

Supplementary Figure 4 Sequence alignment of TAG synthase enzymes of *M. tuberculosis* and *M. smegmatis*. Multiple sequence alignment of Rv3130c protein (*tgs1*) from *M. tuberculosis* and its homologs MSMEG_3948 and MSMEG_5242 from *M. smegmatis* is shown. “^∗^” represents conserved residue in all three cases.

Supplementary Figure 5 TAG accumulation examination in *M. smegmatis* by thin layer chromatography. Panel shows the autoradiogram of the TLC demonstrating the incorporation of radiolabeled ^14^C-oleic acid in TAG in wild-type (WT), *atpD* knock-out (Δ*atpD*), and *atpD*-complemented (Δ*atpD*^*C*^) strains of *M. smegmatis*. Arrowhead points to the various species obtained on TLC such as triacylglycerol (TAG), diacylglycerol (DAG), and monoacylglycerol (MAG). Lane 4 has the free ^14^C-oleic acid. Experiments were repeated at least thrice; only one representative image is shown.

Supplementary Table 1 List of primers used in this study. Primers and their sequences used in the RT-qPCR experiments, and the genes that they target are given.
