# Supplementary material for: Understanding Metabolic Remodeling in Mycobacterium smegmatis to Overcome Energy Exigency and Reductive Stress Under Energy-Compromised State
Source: Front Microbiol. 2021 Sep 1;12:722229. doi: 10.3389/fmicb.2021.722229 (PMC8440910; doi:10.3389/fmicb.2021.722229)
Supplement: Supplementary Figure 1 — Pairwise sequence alignment of WhiB3 protein of M. tuberculosis and M. smegmatis. Pairwise sequence alignment of WhiB3 protein from M. tuberculosis (Rv3416) and MSMEG_1597 from M. smegmatis is shown. “∗” represents conserved residue. [file Data_Sheet_2.pdf]

Supplementary Figures

Figure S1:

```
MsmwhiB3      MPQPQQLPGPNADIWDWQMRGLCRGVDSSMFFHPDGERGRARAQREMRAKEMCRSCPVIA      60
MtbwhiB3      MPQPEQLPGPNADIWNWQLQGLCRGMDSSMFFHPDGERGRARTQREQRAKEMCRRCPVIE      60
              ****:**********:**::*****:****************:*** ******* ****

MsmwhiB3      QCRSHALAVGEPYGIWGGLSESERELLLKRGIRRSA------      96
MtbwhiB3      ACRSHALEVGEPYGVWGGLSESERDLLLKGTMGRTRGIRRTA      102
               ****** ******:*********:****  : *:
```

Figure S1: Pairwise sequence alignment of WhiB3 protein of *M. tuberculosis* and *M. smegmatis*. Pairwise sequence alignment of WhiB3 protein from *M. tuberculosis* (Rv3416) and *MSMEG_1597* from *M. smegmatis* is shown. '*' represents conserved residue.

Figure S2:

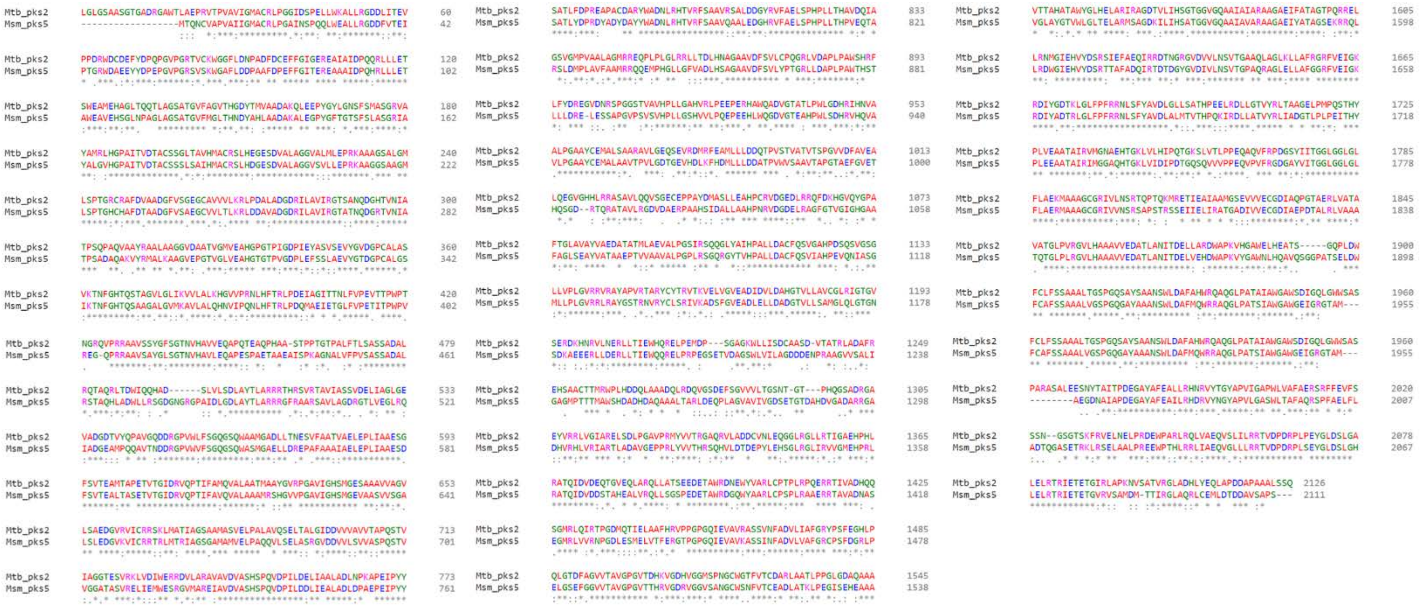

Figure S2. Sequence alignment of polyketide biosynthesis protein of *M. tuberculosis* and *M. smegmatis*. Pairwise sequence alignment of Rv3825c protein (*pks2*) from *M. tuberculosis* and MSMEG_4724 (*pks5*) from *M. smegmatis* is shown. ‘*’ represents conserved residue.

Figure S3:

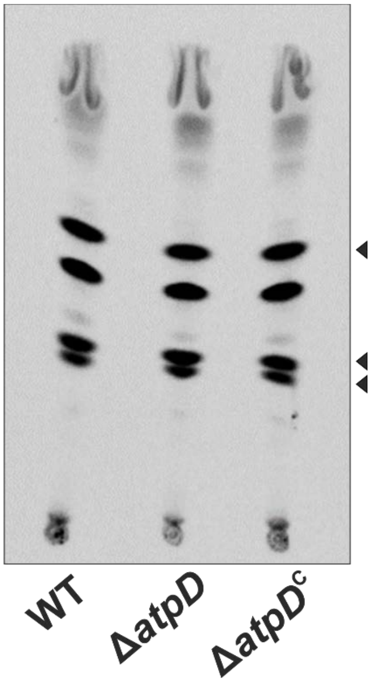

Figure S3. Phospholipid analysis by thin layer chromatography. Panel shows the TLC image depicting the phospholipids in *M. smegmatis* wild-type (WT), *atpD* knock out (Δ*atpD*), and *atpD*-complemented (Δ*atpD*^{C}) strains. Spot corresponding to PE, PI, PS (top to bottom) is marked with arrowhead. Experiments were repeated at least thrice; only one representative image is shown.

Figure S4:

```
MSMEG_3948      ------LSALDVAFLEAEDADRNVSLVIGVLAILEGSPPTDHVLLGSVYNRLMSIPRFTQ    54
Mtb_tgs1        ---MNHLTTLDAGFLKAEDVDRHVSLAIGALAVIEGPAPDQEAFLSSLAQRLRPCTRFGQ    57
MSMEG_5242      MTAPEQLSVLDAGFLEAEDSDPNVSLAIGAVAVMAGPMPDFETLKATLAERILSVPRLRQ    60
                      *:.**..**:*** * :***.**.:*:: *  *  ..: .:: :*:    *: *

MSMEG_3948      IVERQPLDLAAPQWVEAHDFSVAHHVRRTAVPQPGDDTALFGVVADIMERRLDRSRPLWE    114
Mtb_tgs1        RLRLRPFDLGAPKWVDDPDFDLGRHVWRIALPRPGNEDQLFELIADLMARRLDRGRPLWE    117
MSMEG_5242      VLHTRPLDLGAPEWVDDPHLDITRHIRRAALPRPGDDAALHDWVAEVMERRLDRDHPLWQ    120
                 :. :*:**.**:**:  .:.: :*: * *:*:**::  *.  :*::* *****.:***:

MSMEG_3948      CWIIEGLPSDRWAILMKIHHCIADGIAAAQLLSYLSDEGTVDSFSSDIDGAKPTAPQKNR    174
Mtb_tgs1        VWVIEGLADSKWAILTKLHHCMADGIAATHLLAGLSDESMSDSFASNIHTTMQSQSASVR    177
MSMEG_5242      CWVADGLPANRWAILIKIHHCVADGVAAAHLLTRLCDDSPEPSVPHAP-------PPAPR    173
                 *: :**  .:**** *:***:***:**::**: *.*:.   *.              *

MSMEG_3948      RFELTLNPVRLIRSAVDATASVGSEVIRVAEGALQIASGLLDSHPLPLRGPVTDLRRYAS    234
Mtb_tgs1        RGGFRVNPSEALTASTA----VMAGIVRAAKGASEIAAGVLSPAASSLNGPISDLRRYSA    233
MSMEG_5242      QAPFSLNPVDWVTGAWRTAMDVTGIAGKVLHGAGEIVSGLLTPAPSSLTGPVTARRRFAA    233
                :  : :**   : .:      * .    :. .** :*.:*:*      * **::  **:::

MSMEG_3948      TQVSLADVGRICHAYDVTINDVALAAITDSFRAAMIRRGERPGARSLRTLVPVSVRSNDA    294
Mtb_tgs1        AKVPLADVEQVCRKFDVTINDVALAAITESYRNVLIQRGERPRFDSLRTLVPVSTRSNSA    293
MSMEG_5242      AEVSLADVARVCERFDVTVNDVALAAITASFRSMLIARGVAPGPNALPTLVPVSVRPPAG    293
                ::* **** ::*. :***:********* *:*  :* **  *   :* ******.*   .

MSMEG_3948      A-AQVDNRVSLMLPCLPVDIHDPVEQLLTVHRRMENAKRTGQRQAGSVFVSAVNSLPFGI    353
Mtb_tgs1        L-SKTDNRVSLMLPNLPVDQENPLQRLRIVHSRLTRAKAGGQRQFGNTLMAIANRLPFPM    352
MSMEG_5242      AGNDRANQVSVMLPNLPVDQADPVAQLQAVHTRLSKAKASGQRQAGSALVTMAAAVPFPL    353
                     .  *:**:*** ****  :*: :*  ** *: .**  **** *..::: .  :** :

MSMEG_3948      TTLLVRAAVRMPQQSVVTLATNVPGPRQHLKLLGHRVVRVVPIPPIALGLRTGVAILSYA    413
Mtb_tgs1        TAWAVGLLMRLPQRGVVTVATNVPGPRRPLQIMGRRVLDLYPVSPIAMQLRTSVAMLSYA    412
MSMEG_5242      TAWAVRALTRLPQRGVAMLATNVPGPRRRVTILGREVIRLLPVPPIAMRMRTAVAILSYA    413
                *:  *    *:**:.*. :********: : ::*:.*: : *: ***: :**.**:****

MSMEG_3948      DDLVFGITADFDAIPDVEVLADDIQRAVARLARTAELPTRRAPDGTLTVVTTPR- 467
Mtb_tgs1        DDLYFGILADYDVVADAGQLARGIEDAVARLVAISKRRKVTRRRGALSLVV---- 463
MSMEG_5242      DHLAFGIISDYDAEIDVDAVAAGIEQAVARLAQIAVAHIRSTPLGTLALVPGELA 468
                *.* *** :*:*.  *.  :* .*: *****.  :          *:*::*
```

Figure S4. Sequence alignment of TAG synthase enzymes of *M. tuberculosis* and *M. smegmatis*. Multiple sequence alignment of Rv3130c protein (*tgs1*) from *M. tuberculosis* and its homologs MSMEG_3948 and MSMEG_5242 from *M. smegmatis* is shown. '*' represents conserved residue in all three cases.

Figure S5:

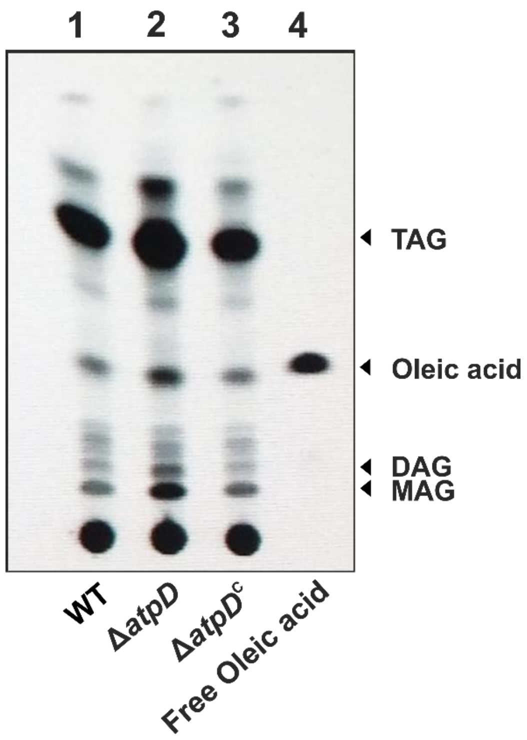

Figure S5. TAG accumulation examination in *M. smegmatis* by thin layer chromatography. Panel shows the autoradiogram of the TLC demonstrating the incorporation of radiolabeled ^{14}C-oleic acid in TAG in wild-type (WT), *atpD* knock-out (Δ*atpD*), and *atpD*-complemented (Δ*atpD*^{C}) strains of *M. smegmatis*. Arrowhead points to the various species obtained on TLC such as triacylglycerol (TAG), diacylglycerol (DAG), and monoacylglycerol (MAG). Lane 4 has the free ^{14}C-oleic acid. Experiments were repeated at least thrice; only one representative image is shown.
