# Supplementary material for: Understanding Metabolic Remodeling in Mycobacterium smegmatis to Overcome Energy Exigency and Reductive Stress Under Energy-Compromised State
Source: Front Microbiol. 2021 Sep 1;12:722229. doi: 10.3389/fmicb.2021.722229 (PMC8440910; doi:10.3389/fmicb.2021.722229)
Supplement: Supplementary Table 1 — List of primers used in this study. Primers and their sequences used in the RT-qPCR experiments, and the genes that they target are given. [file Data_Sheet_1.pdf]

Supplementary Table

Table S1: List of primers used in this study. Primers and their sequences used in the RT-qPCR experiments, and the genes that they target are given.

Label	Sequence (5'-3')	Gene
pgsA1_frw	GTGGTCGTCTCGTTCTTCGTGC	*pgsA1*
pgsA1_rev	CTGTGAGGTGACCAGGCAGATC	
pgsA2_frw	GTGTTCCTGTACCTGCTGCTGGTC	*pgsA2*
pgsA2_rev	GACGGTCACCATGTAGATGCGATC	
pgsA3_frw	GTGTGGATCTACCAGTTCAAGATCTCG	*pgsA3*
pgsA3_rev	GCATCACGAGTTCGTTGAACCGGTCG	
glpk_RT_frw	ATGCTGATGGATCTGGAGACCCTCG	*glpK*
glpk_RT_rev	GGTGTTGAGCAGCAGGAAGTTGC	
GPAT_RT_frw	GTTGCTGGGTCCGGAATACAAGTC	*GPAT*
GPAT_RT_rev	CTCGATGCGGTCGTAGTCGATTCG	
AGPAT_RT_frw	GTTCAAGCAGAGCCTGCGGGACAG	*AGPAT*
AGPAT_RT_rev	GACTCGTCGGTGATCCGGTCAC	
WSDGAT1_RT_frw	GACCACAAGCTCGACGATTACC	*WSDGAT1*
WSDGAT1_RT_rev	GAATCGAGTTCCTCGTCGGGGTAG	
WSDGAT2_RT_frw	ATGCAGTCACCGATGGACATCACC	*WSDGAT2*
WSDGAT2_RT_rev	GGTGAGAGATCCTGCGGGATG	
5242_RT_frw	GCTGGGCGATCCTGATCAAGATCCAC	*MSMEG_5242*
5242_RT_rev	CAGTCGACGGGGTTGAGCGAGAAC	
3948_RT_frw	GCACCGCAGAAAAATCGACGCTTCG	*MSMEG_3948*
3948_RT_rev	GATGCGTATCGGCGCAGATCCGTCAC	
PE_RT_frw	CATGCCGCTCAAATGTGCCGGTAG	*PE*
PE_RT_frw	CATGCCGCTCAAATGTGCCGGTAG	
PS_RT_frw	GTGTGGCACCTGCGTTCATCGTGTAC	*PS*
PS_RT_rev	CACGTCCAGCAGCGCGTTGAACC	

PI_RT_frw	GTGGTCGTCTCGTTCTTCGTGCTC	*PI*
PI_RT_rev	GATGTAGGAGATCACCTGTGAGGTGAC	
lipG_RT_frw	GTGCGCAGATGCTGATGTGGCGTAC	*lipG*
lipG_RT_rev	GACACGCTGCCCCGACAGTTTC	
lipJ_RT_frw	GAGGAGTTCGTCACGGGAAACCATGC	*lipJ*
lipJ_RT_rev	CAGTTCCTCGGTGACCTCACCGAAC	
lipI_RT_frw	GATCGATGCCGTCAAGGGCTTCTC	*lipI*
lipI_RT_rev	CAGCGTGTCGGCGTTGTCCAAC	
lipN_RT_frw	GTTCGTCATAGGTGATCTGGAGGTC	*lipN*
lipN_RT_rev	CGTGTTCGAGTGCCCACTGATAGG	
lipO_RT_frw	GGTGAAGGCCAACATCGCCGACTTC	*lipO*
lipO_RT_rev	GAGCCGCTCCAGGATCTCCATGAACTC	
lipT_RT_frw	GAAGAACTCATCAACCGTGGCATGACG	*lipT*
lipT_RT_rev	GCATCGGCGAACAGCTTCTCGATCATC	
lipZ_RT_frw	CTGATGCAGAAGGTCATGTCGCGAAC	*lipZ*
lipZ_RT_rev	GACGGCTTGGTGTTCACTCCGAAC	
WhiB3_RT_frw	GTGCGAAGGAAATGTGTCGCAGTTG	*WhiB3*
WhiB3_RT_rev	GCAGAAGCTCACGCTCGGATTC	
Msm_4727_RTfrw	CTCACTCGAAGACGGCGTGAAG	*pks5*
Msm_4727_RTrev	CACATCTCGATCAGTTCGCGCAC	
Msm_0408_RTfrw	CGAGTTGGATGGTGACACCGAATC	*pks1*
Msm_0408_RTrev	ACTCGTCGGTGAGGCGCTC	
Msm_6398_RTfrw	CAGCTTCTACAGCGACTGGTACAAG	*fbpA*
Msm_6398_RTrev	GAAAGCGATGCCGCGTAGATGAAC	
Msm_2078_RTfrw	CACCAACGCGTTCGAGATGTTCC	*fbpB*
Msm_2078_RTrev	CTGGTCAGGAAGGTCTCCCAC	
Msm_4731_RTfrw	CTGACGGCGAACTTCGAACAGG	*fadD28*
Msm_4731_RTrev	GTCGCCAGAAGCTGCATCCATC	
Msm_4728_RTfrw	ATCGTGCGGCGAAAGTTACGCAG	*los*
Msm_4728_RTrev	CAGCGCCGCGTACATCAGG	

Msm_4741_RTfrw	CTCGACAAGCACTTCCCGGTG	*MmpL*
Msm_4741_RTrev	AGGGTGGACGCGTCATTGAGC	
accA3_RTfrw	CTCTCCGAGAACGCCGACTTC	*accA3*
accA3_RTrev	CACACCGTGCTCCTTGGCGAAC	
accD4_RTfrw	GCAACATCCACAAGGTGGTCAACTC	*accD4*
accD4_RTrev	GCAGGATCTCGTGCATGTCGTAC	
accD6_RTfrw	GACGTCGACATGGTGTCGCTC	*accD6*
accD6_RTrev	CTGCTGGCAGAACAATCCGACCAG	
FAS1_RTfrw	CTACCTGTGGAAGCTGCAGGTC	*FAS1*
FAS1_RTrev	CGTTGAGTTCGTCGATGATGTCGAC	
Ndh_RT_frw	GCAAGCAGAAGAACGCCCAGG	*ndh*
Ndh_RT_rev	CTTCATGCCGGGTGCGAACTC	
NuoA_RT_frw	GTCGAAACTCGAAGCCTACGAATGC	*nuoA*
NuoA_RT_rev	GATCTCGATGTCGAAGACGATGAACAAC	
NuoB_RT_frw	CTGCGCGATCGAGATGATGTCAAC	*nuoB*
NuoB_RT_rev	CCATTTCGGTTCGACCATCTGGTC	
NuoC_RT_frw	GGAACGGTACGCGATGTCGATC	*nuoC*
NuoC_RT_rev	GACACCCAGGCACAGTTCGAAG	
NuoD_RT_frw	GCTCAACGAGAACTACATCTGGAAGG	*nuoD*
NuoD_RT_rev	GCATCTCCTTGACCCGGATGATG	
NuoE_RT_frw	GTCCAGGCACAGGACGGATATC	*nuoE*
NuoE_RT_rev	CACTCGACGTGTTCTAACGTCACC	
NuoF_RT_frw	CAGGCACGCGTTCATCTACGTG	*nuoF*
NuoF_RT_rev	GTCTCCTCACCGCAGATGTACG	
NuoG_RT_frw	CAAACCGTTCCAGTCCTACTTCTCC	*nuoG*
NuoG_RT_rev	CAGTTCCACTCCTCGTTCACCTC	
qcrB_RT_frw	GCTGCTGATCCTGGCGATGTTC	*qcrB*
qcrB_RT_rev	CAGACGCGGGATCAGGATCTC	
qcrC_RT_frw	CGTGTACTTCCAGGTGTCGACC	*qcrC*
qcrC_RT_rev	GCAGTTCAGGCGGAACAGGTC	

ctaC_RT_frw	GCTGTGGATCGGTTCCGTGATTG	*ctaC*
ctaC_RT_rev	GGTGAAGTAGAACAGCACCGAGATG	
ctaD_RT_frw	GTGCGGTCAACATGATCACCACG	*ctaD*
ctaD_RT_rev	CGAAGAACCAGAACAGGTGCTCC	
CydA_RT_frw	GAGATGTACGCCTCGATGTTCTCG	*cydA*
CydA_RT_rev	CAACGCGAAGATGCTCTCCAATTCG	
CydB_RT_frw	GACCACAAGCTCGACGATTACC	*cydB*
CydB_RT_rev	GGATCTGCTTGTCGGCATCGAC	
CydD_RT_frw	CATCCCGATCTTCATGGTGCTGATC	*cydD*
CydD_RT_rev	GCAATGTGGCCATCGTCGAACG	
Msm_4325_RTfrw	GTTCCACACCCACTACATGGCATCG	*fabD*
Msm_4325_RTrev	GGTGAGTTGTGCGACGAGCTTCTC	
Msm_4327_RTfrw	GCATGTCGAAGTTCCTCAGCAAGC	*kasA*
Msm_4327_RTrev	CGTACGTCTCGACGATCTTCTCG	
Msm_4328_RTfrw	GAGAGCAAGGCAATCAACAATGCGATGG	*kasB*
Msm_4328_RTrev	CGATTTCCGGATCCAGGTTGTTCAGG	
Msm_3150_RTfrw	GTGTTCAATGTGACGTCACCGACAGC	*mabA*
Msm_3150_RTrev	GGTTGGTGTTGATGACCTCTTCGAACC	
